# The Effects of Functionally Guided, Connectivity-Based rTMS on Amygdala Activation

**DOI:** 10.3390/brainsci11040494

**Published:** 2021-04-13

**Authors:** Lysianne Beynel, Ethan Campbell, Maria Naclerio, Jeffrey T. Galla, Angikar Ghosal, Andrew M. Michael, Nathan A. Kimbrel, Simon W. Davis, Lawrence G. Appelbaum

**Affiliations:** 1 Department of Psychiatry and Behavioral Science, Duke University School of Medicine, 200 Trent Drive, Box 3620 DUMC, Durham, NC 27710, USA; ecamp2016@gmail.com (E.C.); maria.naclerio@duke.edu (M.N.); jeffrey.galla@duke.edu (J.T.G.); angikar.ghosal@duke.edu (A.G.); nathan.kimbrel@duke.edu (N.A.K.); greg@duke.edu (L.G.A.); 2 Duke Institute for Brain Sciences, Duke University School of Medicine, 200 Trent Drive, Box 3620 DUMC, Durham, NC 27710, USA; andrew.michael@duke.edu (A.M.M.); simon.davis@duke.edu (S.W.D.); 3 Durham Veterans Affairs (VA) Health Care System, 508 Fulton St, Durham, NC 27705, USA; 4 VA Mid-Atlantic Mental Illness Research, Education, and Clinical Center (MIRECC), 3022 Croasdaile Drive, Durham, NC 27705, USA; 5 Department of Neurology, Duke University School of Medicine, 3116 N Duke Street, Durham, NC 27704, USA; 6 Center for Cognitive Neuroscience, Duke University, 308 Research Drive, Durham, NC 27710, USA

**Keywords:** repetitive transcranial magnetic stimulation, amygdala, fMRI, functional connectivity

## Abstract

While repetitive transcranial magnetic stimulation (rTMS) is widely used to treat psychiatric disorders, innovations are needed to improve its efficacy. An important limitation is that while psychiatric disorders are associated with fronto-limbic dysregulation, rTMS does not have sufficient depth penetration to modulate affected subcortical structures. Recent advances in task-related functional connectivity provide a means to better link superficial and deeper cortical sources with the possibility of increasing fronto-limbic modulation to induce stronger therapeutic effects. The objective of this pilot study was to test whether task-related, connectivity-based rTMS could modulate amygdala activation through its connectivity with the medial prefrontal cortex (mPFC). fMRI was collected to identify a node in the mPFC showing the strongest connectivity with the amygdala, as defined by psychophysiological interaction analysis. To promote Hebbian-like plasticity, and potentially stronger modulation, 5 Hz rTMS was applied while participants viewed frightening video-clips that engaged the fronto-limbic network. Significant increases in both the mPFC and amygdala were found for active rTMS compared to sham, offering promising preliminary evidence that functional connectivity-based targeting may provide a useful approach to treat network dysregulation. Further research is needed to better understand connectivity influences on rTMS effects to leverage this information to improve therapeutic applications.

## 1. Introduction

Repetitive transcranial magnetic stimulation (rTMS) is a non-invasive brain stimulation approach that uses rapidly changing magnetic fields to modulate neuronal activity underneath a stimulating coil. rTMS is approved by the U.S. Food and Drug Administration as a therapy for treatment-resistant depression and obsessive compulsive disorders and has also been proposed as a potential treatment for patients with posttraumatic stress disorders (PTSD). As reported in a recent comprehensive review [1], 13 studies have been published that attempt to test rTMS effects on PTSD symptoms and/or pathophysiology. Collectively, these studies demonstrate significant, but modest PTSD symptom improvements from rTMS treatment. Notably, this literature includes substantial variability in rTMS protocols (e.g., they use inhibitory [2,3,4,5,6,7,8,9,10,11], excitatory [2,5,10,12,13,14,15], or a combination of both inhibitory and excitatory pulse sequences [16]; as well as stimulation of both the right e.g., [4] and left cortical hemispheres e.g., [14]); however, despite these substantial differences, these protocols have generally produced equivalent effects on PTSD symptom improvement. One possible explanation for this equivalency is that rTMS may propagate across multiple brain regions involved in PTSD pathophysiology, with differing influences at different nodes of this network. Indeed, while rTMS effects are often assumed to be relatively focal and superficial, with a depth penetration to about 2 cm below the scalp [17], recent neuroimaging studies demonstrate modulation of interconnected brain regions [18], including deep brain structures, which lie well beyond the spread of the magnetic field [19]. In a systematic review of 33 studies with baseline and post-rTMS measures of fMRI resting-state functional connectivity, it has been found that rTMS can induce significant changes in brain connectivity that spread both within and between functional brain networks, and produce a diverse pattern of connectivity increases and decreases within these networks [20].

In light of rTMS effects on fMRI blood oxygen level dependent (BOLD) signal and functional connectivity, recent studies are attempting to indirectly target distal brain areas through their resting-state functional connections with accessible, proximal cortical areas. This approach has been used successfully to modulate hippocampus [21], insula [22,23] and amygdala [24]. Such studies have demonstrated that “connectivity-based” rTMS may provide a promising approach to modulate deep brain regions, which is highly relevant when using rTMS as a treatment for psychiatric disorders that are hypothesized to primarily stem from fronto-limbic dysfunction.

In the current study, we explore these possibilities by implementing task-related functional connectivity-based rTMS to test for BOLD and connectivity changes due to stimulation. For this purpose, we derive individualized rTMS targets through application of psychophysiological interaction (PPI) analysis, in order to determine a location in the medial prefrontal cortex (mPFC) that shows maximal negative functional connectivity with the right amygdala, as participants passively viewed frightening or neutral pictures. Using this target, 5 Hz rTMS was applied “online”, as participants concurrently watched frightening video clips to engage the fronto-limbic network and induce Hebbian-like plasticity [25]. Based on the common frequency dependent heuristic of rTMS [26], it was expected that such excitatory stimulation to a negatively connected mPFC node would strengthen negative connectivity with the amygdala and consequently inhibit amygdala activity. Moreover, through Hebbian-like plasticity mechanisms it was expected that subjects experiencing the strongest feelings of fear, as reflected by changes in heart rate collected during the video-clips, would also show the strongest rTMS-induced changes in amygdala activation. To test the specificity of these effects resting-state fMRI scans were also collected before and after the rTMS intervention.

## 2. Materials and Methods

### 2.1. Participants

**Figure 1:**
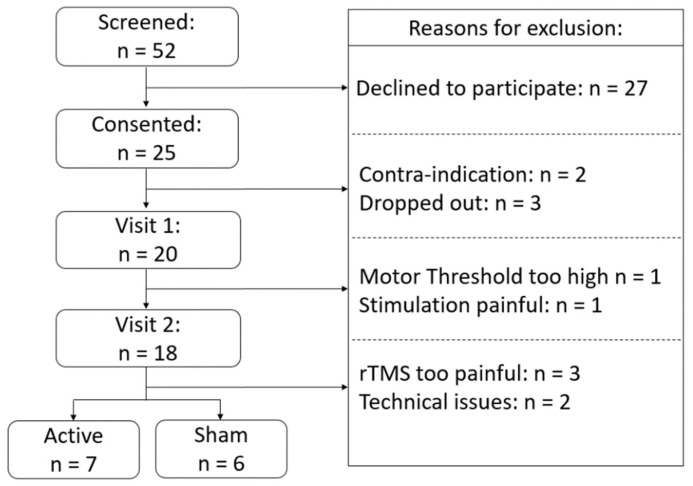
Consort diagram showing the recruitment, exclusion and inclusion numbers.

Fifty-two healthy young adults (18–35 years old) were recruited through online advertisements; flyers posted on campus; or an existing subject pool maintained through the university neuroimaging center to participate in this this single blind, randomized sham-controlled, two-visit study. The study was pre-registered on ClinicalTrials.gov (NCT03746405), and approved by the Duke University Health System Institutional Review Board (#Pro00101172). After the first phone screen, 27 participants declined to participate, and 25 were included in the study. During the first visit, participants completed an eligibility screening to ensure they did not have any contraindication to TMS (TASS [27]) or to MRI, followed by a psychiatric screening using the Mini-International Neuropsychiatric Interview [28] to ensure they did not have any disqualifying psychiatric disorders. They were then asked for a urine sample to ensure that the participants were not under the influence of any substances that could lower their seizure threshold and were not pregnant. Participants who made it through the above screenings (*n* = 23) were included in the study. Subsequently, three participants withdrew from the study due to scheduling conflicts, resulting in 20 participants completing the first visit (see Figure 1 for consort diagram). Of the twenty participants who underwent rMT assessment, one was excluded because reported discomfort and a second was excluded because their rMT was above 83% of maximum stimulator output (MSO) and therefore at 120% of rMT would have exceeded the possible device output during the rTMS session. The remaining eighteen participants were randomized into either an active or sham rTMS group and contacted to participate in the second visit. Although a slow ramp-up procedure was used to acclimate participants to the sensation of rTMS, stimulation was still too uncomfortable for three participants who withdrew from the study. Two additional participants were excluded because of technical issues during the MRI acquisition that caused unanticipated data loss. The final analyzed sample for this study, therefore included thirteen participants, seven receiving active rTMS and six receiving sham stimulation.

### 2.2. Experimental Procedure

**Figure 2:**
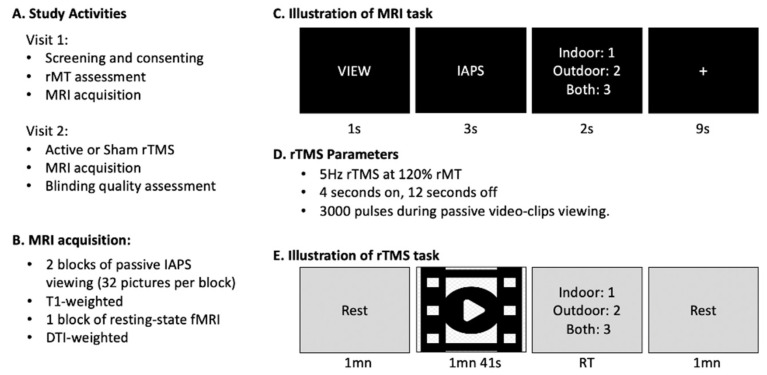
(**A**) Activities done during each study visit. (**B**) Scans during MRI acquisition. (**C**) Illustration of MRI task showing passive viewing of IAPS pictures. (**D**) rTMS parameters. (**E**) Illustration of rTMS task showing passive viewing of frightening video-clips from the Schaefer et al. database. In order to maintain attention in both the MRI and rTMS tasks, participants were asked to rate whether the scene was indoor, outdoor, or both.

This study consisted of two experimental visits (Figure 2A). On the first visit, following screening and consenting, resting motor threshold (rMT) was determined and an MRI session was conducted to locate each participants’ rTMS target. During the second visit, which occurred within a week of the first visit, online rTMS was applied to individualized target locations as subjects viewed emotionally arousing video clips, immediately followed by a second MRI acquisition. rTMS effects were assessed by comparing fMRI BOLD signal and functional connectivity changes between these two visits.

#### 2.2.1. Resting Motor Threshold (rMT)

rMT was performed with an active/placebo figure-8 coil (A/P Cool-B65) and a MagPro X100 stimulator with MagOption (MagVenture, Denmark), while the coil position was continually monitored through a stereotaxic neuronavigation system (Brainsight, Rogue Research, Canada). To define rMT, electrodes (Neuroline 720, Ambu, Columbia, MD, USA) were placed on the right first dorsal interosseous (FDI) muscle in a belly–tendon montage and motor evoked potentials (MEPs) were recorded through the neuronavigation system. The motor “hot spot” was defined as the position over the left motor cortex that elicited the greatest MEP in the right FDI. rMT was then defined as the TMS pulse intensity producing 50 μV peak-to-peak MEP amplitude, using a maximum likelihood estimator. (TMS Motor Threshold Assessment Tool, MTAT 2.0, [29]).

#### 2.2.2. MRI Acquisition

Participants completed a 30-min MRI session (Figure 2B) that included a T1-weighted anatomical image (3D-T1-weighted echo-planar sequence, acquisition matrix = 256 mm^2^, time repetition [TR] = 7148 ms, time echo [TE] = 2.7 ms, field of view [FOV] = 256 mm^2^, spacing between slices = 1 mm, 196 slices), and a diffusion tensor imaging scan (acquisition matrix = 256 mm^2^, TR = 17,000 ms, TE = 91.4 ms, FOV = 256 mm^2^, spacing between slices = 2 mm, b-value = 2000 s/mm^2^, diffusion-sensitizing directions = 25). Three runs of coplanar EPI functional images were also acquired with an oblique axial orientation including a resting-state scan and two blocks of passive viewing of IAPS pictures using the same acquisition parameters: (acquisition matrix = 128 mm^2^, TR = 2000 ms, TE = 25 ms, spacing between slices = 4 mm, 240 volumes = 8 min per block). MRI acquisitions were collected on the first visit and on the second visit after rTMS intervention.

Each fMRI task block consisted of 32 trials. On each trial, a “VIEW” instruction was displayed on the screen for one second, followed by three seconds of a picture from the International Affective Picture System, IAPS, [30]. Three types of emotional pictures were used: fearful (50% of the trials), neutral (40%), and happy (10%). The images that were selected have been found to effectively evoke the desired affective responses [31,32]. The “happy” category was added to reactivate the fronto-limbic network and limit habituation. In order to ensure that participants paid attention to the stimuli, they were asked to perform, in less than 2 s, a shallow scene judgment by indicating whether the image presented was outdoor, indoor or both, by pressing 1, 2 or 3 on the button box (see Figure 2C). Finally, a fixation cross was presented for nine seconds to allow the hemodynamic response to return to baseline, before the start of the next trial. To have a shorter delay between the end of rTMS and our task of interest, the functional task was always performed first and followed by the anatomical scan. During the resting-state acquisition, participants were asked to keep their eyes opened and to look at a white fixation cross on a black background. This setup has been shown to produce high test–retest reliability [33]. During the functional scan, visual stimuli were back projected onto a screen located at the foot of the MRI bed using an LCD projector. Subjects viewed the screen via a mirror system located in the head coil and the start of each run was electronically synchronized with the MRI acquisition computer. Behavioral responses were recorded with a 4-key fiber-optic response box (Resonance Technology Inc., Northridge, CA, USA). Scanner noise was reduced with ear plugs, and head motion was minimized with foam pads. When necessary, vision was corrected using MRI-compatible lenses that matched the distance prescription used by the participant.

#### 2.2.3. MRI Processing for Targeting Approach

Following the first visit, functional connectivity between the right amygdala and medial prefrontal cortex (mPFC) was determined using psychophysiological interaction (PPI) analysis. To do so, functional images were skull stripped, reoriented and corrected for slice acquisition timing, motion, and linear trend using the FMRIB Software Library (FSL) [34]. Motion correction was performed using FSL’s MCFLIRT, and six motion parameters were then regressed out of each functional voxel using standard linear regression. Images were then temporally smoothed with a high-pass filter using a 190s cut off and normalized to the Montreal Neurological Institute (MNI) stereotaxic space.

Separate events were modeled for the viewing of the instructions (duration: 1s), rating (duration: 2s), and each of the emotional picture categories (fear, happy, neutral; duration: 3s), all with an onset at the beginning of the event, as recorded by the Matlab script used to launch the MRI acquisition. At the first level, functional data were analyzed as individual runs, using a general linear model (GLM) in which trial events were convolved with a double-gamma hemodynamic response function. The Fear > Neutral contrast was generated, allowing the identification of individualized statistical maps showing stronger BOLD activity when participants were seeing the fearful compared to the neutral pictures.

PPI analysis was then performed, for each functional run, following the FSL-PPI pipeline [35]. The design file from the previous BOLD analysis was used to generate the task regressor. To extract the time course of the right amygdala, the “fslmeants” command was run by using the filtered functional data as the input and the right amygdala mask (ROI # 42) as defined by the AAL16 atlas [36]. These two events were then loaded into FSL: the task regressor as the psychological regressor, and the time course of the amygdala as the physiological regressor. The third event, the PPI was generated as the interaction between the task regressor and the amygdala. The remaining task regressors (happy, view and rate) from the original BOLD analysis were also included. The second level analysis was then performed to collapse information from both runs using a fixed-effects model. The subsequent statistical map was then moved back from MNI space to individual space using a linear registration (FLIRT). This map was then overlaid on the anatomical image on the neuronavigation software, and the region within the mPFC mask showing the strongest negative z-value was defined as the TMS target.

#### 2.2.4. Online rTMS Procedure

During the second session, 5 Hz rTMS was applied at 120% rMT over the mPFC target location, with the TMS coil handle was pointing upward. Stimulation was applied for 40 min, in trains of 4 s separated by inter-train intervals of 12 s (Figure 2D). These parameters replicate the ones used by Philip et al. [37] who demonstrated significant connectivity changes between amygdala and mPFC in patients with posttraumatic stress disorder. Each participant received either active or somatosensory-matched sham stimulation, with the random allocation and assignment defined after each participant’s first visit. Sham stimulation was applied using the same coil in placebo mode, which produced similar clicking sounds and somatosensory sensation (via electrical stimulation with scalp electrodes) as the active mode, but with a greatly attenuated magnetic field that was shielded from the skull. To increase tolerability, lidocaine cream was applied on participant’s forehead before starting the experiment; and a ramp-up procedure was used by starting the stimulation at a very low intensity (10% MSO) and increasing it by 5% step during each rTMS inter train interval.

Given the importance of state-dependency on rTMS effect [38] and in order to promote Hebbian-like plasticity, the stimulated fronto-limbic network was engaged during rTMS through the passive viewing of frightening movie clips that have been shown to reliably and effectively evoke feelings of fear [39]. Each video-clip was followed by a shallow scene judgment (indoor, outdoor or both). Before the first movie clip and between the subsequent clips, there was a one-minute period where participants were instructed to rest (Figure 2E). Electrocardiography (ECG) was acquired throughout this task (LabChart, ADInstrument, Sydney, Australia).

#### 2.2.5. MRI Processing for Group Analysis to Assess rTMS Effect on Amygdala Activation and Connectivity Changes

Analysis of the rTMS effects on amygdala activation during passive viewing of IAPS pictures were done to emphasize emotional content of the images. As such, “VIEW” events were not included, and the duration of the picture event was increased from 3 to 6 s, therefore including the rating event, and allowing a better representation of the hemodynamic response to implicit emotional processing of the image. The happy and neutral IAPS pictures were collapsed together and labeled as the “other” emotion which were contrasted with the fearful IAPS images. To assess connectivity changes, the “fear versus other” contrast was extracted from the BOLD analysis and used as the task regressor. The time course of the right amygdala was extracted as the physiological regressor. The third event, the PPI was generated as the interaction between the task and physiological regressor. No other events were entered into the analysis. For each of these outcomes, a 2*2 ANOVA was conducted with Timing (Visit 1 and Visit 2) as the within-subject factor and Stimulation (Active or Sham rTMS) as the between-subject factor.

For the resting-state analysis, the same pre-processing steps used for task-related acquisitions were performed. The time series from these preprocessed images were then extracted from the two ROIs of interest: the left mPFC and the right amygdala. The correlation between these ROIs were calculated for resting-state acquisitions collected before and after rTMS and Fisher r-to-z transformations were used to test to assess the significance of the difference between the correlation coefficients.

#### 2.2.6. Blinding Quality Assessment

At the end of the second visit, to assess the quality of stimulation blinding, participants were asked to guess whether they received active or sham rTMS, and to rate their confidence in their guess on a scale from 0, indicating that they are not confident at all, to 100 indicating high confidence.

#### 2.2.7. ECG Processing

To test whether participants expressed physiological fear responses while passively watching the video clips, heart rate beats per minutes (BPM) and heart rate variability (HRV) were extracted from the ECG data for each movie and rest periods. BPM and HRV for the nine movies and resting periods were averaged separately. An ANCOVA was then performed between the Stimulation (Active or Sham) and the Condition (Movies and Rest), with data from the first resting period, acquired before rTMS, used as the covariate to control for individual differences. According to the state-dependency assumption, individuals benefiting the most from rTMS should be the ones most engaged in the task, as indicated by their physiological responses. In this study, the changes in BPM and HRV between movies and resting periods were used as indicator of engagement in the task, and correlated with changes in amygdala activation between the two visits.

## 3. Results

### 3.1. Tolerability and Blinding

As mentioned in the method section and in Figure 1, although rTMS intensity was calibrated according to rMT for each individual and a slow ramp-up procedure was used to acclimate participant to the sensation, stimulation was still too uncomfortable for three participants who withdrew from the study. As such, 13 subjects (7 females and 6 males) completed the full protocol and were included in the final analysis. These individuals had a mean age of 23.6 years (SD = 3.01), with seven participants randomly assigned to active group and six to the sham group.

To assess the quality of the blinding process a chi-square test of independence was performed. This test did not reveal any dependence between participants’ actual and guessed group assignments (*p* = 0.42). However, a significant relationship was found between the confidence of their guess, and the true delivered stimulation (*p* < 0.05). The numerical values (Table 1) indicate that participants tend to guess that they received active stimulation for both the active and sham true stimulation condition, which support a good blinding quality.

### 3.2. MRI and Electrophysiological Changes

#### 3.2.1. BOLD Changes in Fearful Versus Other Contrast

**Figure 3:**
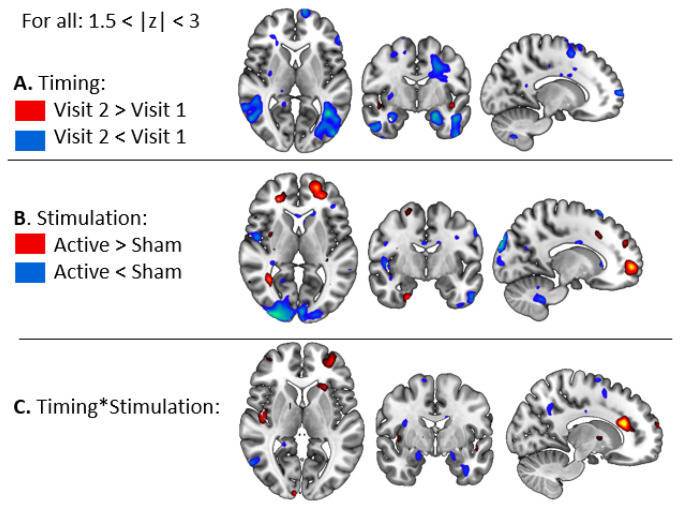
Results from the 2*2 ANOVA on BOLD signal. (**A**) Main effect of Timing (Pre versus Post rTMS). (**B**) Main effect of Stimulation (Active versus Sham rTMS), and (**C**) Interaction between Timing and Stimulation.

Results from the ANOVA revealed a main effect of Timing with significantly less activations on Visit 2 than on Visit 1, in particular in the bilateral amygdala (Figure 3A). Results also showed a main effect of Stimulation, with stronger activation for Active than Sham rTMS in the prefrontal cortex (Figure 3B), and a significant interaction between Timing and Stimulation (Figure 3C).

**Figure 4:**
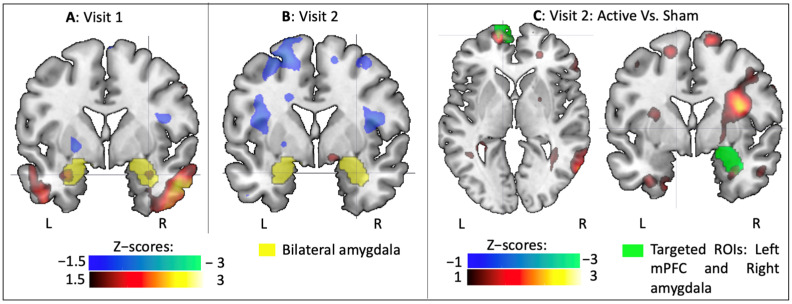
BOLD signal analysis with for the Fear versus Other contrast on Visit 1 (**A**), Visit 2 (**B**), and comparison of the effects of Active versus Sham rTMS on the second visit (**C**). Heat maps indicate Z-scores, blue colors indicate a decrease in BOLD signal and red colors indicate increase. Yellow shading represents the amygdala; and green shading represents masks used to define left mPFC and right amygdala.

Since significant differences were found in the main analysis, subsequent t-tests were performed to better assess each pairwise contrast. First, BOLD signal in the Fear versus Other contrast was computed in each visit separately. During the first visit, the presentation of frightening IAPS pictures increased amygdala activation when compared to other pictures (Figure 4A). This result demonstrated that the task induced expected affective brain changes and allowed for investigation of subsequent amygdala changes after rTMS. During the second visit, it was found that the amygdala was no longer significantly activated (Figure 4B), likely due to habituation, which has previously been reported in similar studies [40], and likely explains the main effect of timing found in the omnibus analysis. The interaction was then decomposed to assess the influence of stimulation condition, during Visit 2. Given the limited number of subjects in this analysis (7 versus 6) the significance threshold level was decreased from 1.5 to 1. Here, it was found that when compared to subjects receiving electrical sham stimulation, subjects receiving active rTMS displayed stronger activation both in the stimulated left mPFC, and in the indirectly targeted right amygdala (Figure 4C, green shaded ROIs). This finding indicates that active rTMS, applied over the mPFC counteracts habituation and provides evidence that connectivity-based rTMS is able to modulate amygdala, in a manner that is specific to the hemisphere of stimulation.

**Figure 5:**
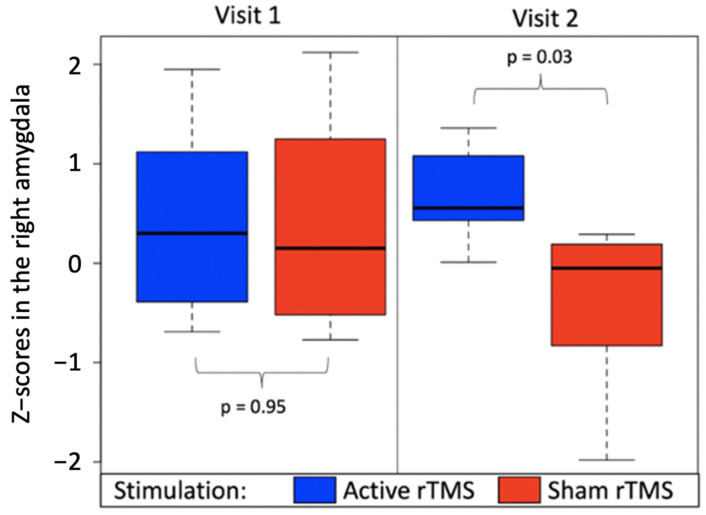
fMRI activation in the right amygdala (z-scores) obtained before rTMS (Visit 1) and after rTMS (Visit 2) for subjects receiving active stimulation (blue) or sham stimulation (red). The *p*-values are reported for independent t-tests comparing amygdala activation in each group, within each visit.

To more specifically test the effect of rTMS on BOLD activity in the right amygdala, an ROI analysis was performed. The right amygdala ROI was first generated by combining the group activation from Visit 1 (z > 1.5) and the anatomical mask from the AAL atlas. Z-scores in the Fear versus Other contrast within this ROI were then extracted for all subjects at each visit. Two independent t-tests were then performed on these z-scores to investigate the differences between the two groups. While, as expected, no differences were found on the first visit (*p* = 0.95), a significant difference was found on the second visit, with subjects in the active group showing significantly greater amygdala activation (mean = 0.67, standard deviation = 0.48) than subjects receiving sham rTMS (mean = −0.48, standard deviation = 0.87; *p* = 0.03) (Figure 5). This ROI analysis therefore confirmed the results from the whole-brain analysis with active rTMS significantly increasing activity in the right amygdala compared to sham rTMS.

#### 3.2.2. Task-Related Functional Connectivity Change

**Figure 6:**
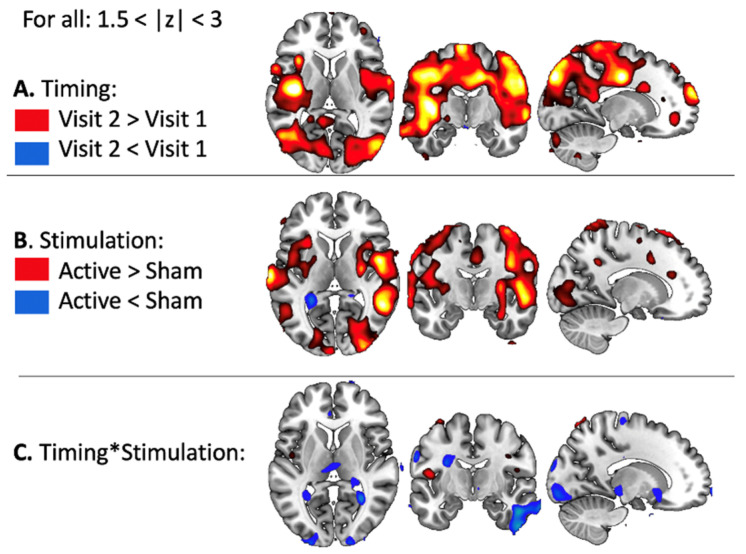
Results from the 2*2 ANOVA on task-related functional connectivity. (**A**) Main effect of Timing (Pre versus Post rTMS). (**B**) Main effect of Stimulation (Active versus Sham rTMS), and (**C**) Interaction between Timing and Stimulation.

Results from the ANOVA revealed a main effect of Timing with significantly stronger connectivity between the right amygdala and the whole brain on Visit 2 than on Visit 1 (Figure 6A). A main effect of Stimulation was also found with stronger connectivity after Active compared to Sham rTMS (Figure 6B) and a significant interaction between Timing and Stimulation (Figure 6C).

**Figure 7:**
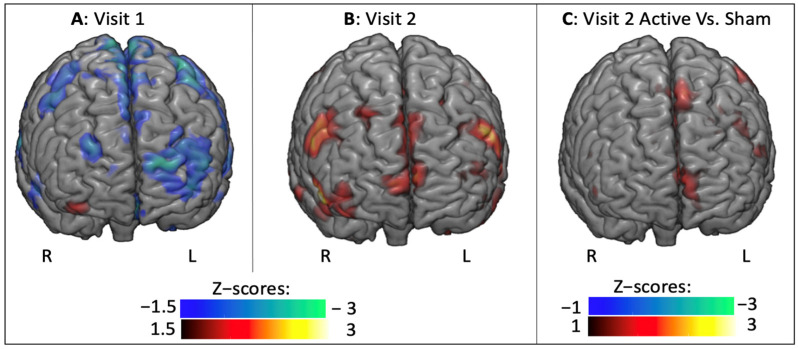
PPI analysis for the Fear versus Other contrast on Visit 1 (**A**), Visit 2 (**B**), and comparison of the effects of Active versus Sham rTMS on the second visit (**C**). Blue colors indicate decreased connectivity; and red colors indicate increased in functional connectivity.

To better understand these effects, t-tests were performed on each visit separately. These analyses demonstrated that the connectivity pattern was reversed during the second visit, by switching from a negative connectivity between the right amygdala and the whole brain, during the first visit (Figure 7A) to a positive connectivity on the second visit (Figure 7B). When comparing the effects of Active and Sham rTMS in Visit 2, it was observed that these changes were due to increased functional connectivity from Active rTMS to the stimulated left mPFC (Figure 7C).

#### 3.2.3. Resting-State Functional Connectivity Change

The Fisher r-to-z transformations performed to assess changes in ROI-based resting-state connectivity between the left mPFC and the right amygdala did not reveal any significant changes with sham rTMS (Visit 1: r = 0.44; Visit 2: r = 0.44, z = 0, *p* = 1) or active rTMS, even though the correlation strengthened numerically following active stimulation (Visit 1: r = 0.44, Visit 2: r = 0.49; z = −0.09, *p* = 0.92). This lack of difference could be explained by the limited sample size, or because resting-state was collected at least 20 min after the end of the rTMS application (after two blocks of task and an anatomical scan acquisition), possibly too late to observe residual post-effects of stimulation.

#### 3.2.4. Beats per Minute and Heart Rate Variability during Movies and Resting Periods

Two mixed measures ANCOVAs were used to assess changes between the frightening movies and the rest condition, and to test the effect of Stimulation on BPM and HRV, using the first resting period as a covariate. Contrary to expectations, no significant differences were found on the BPM between the two conditions of interest (Movie: 67 ± 11.14 versus Rest: 66.28 ± 10.82, F(1,12) < 1). No differences were found between Stimulation (Active: 65.29 ± 12.32 versus Sham: 67.82 ± 9.52; F(1,12) < 1), and the interaction between these two factors was not significant (F(1,12) = 1.25, *p* = 0.29) (see Table 2 for numerical values). The same pattern of null results were found for HRV, with no main effects of Condition (Movie: 0.93 ± 0.18 versus Rest: 0.95 ± 0.18, F(1,12) < 1) or Stimulation (Active: 0.95 ± 0.18 Sham: 0.92 ± 0.17), or interaction between Stimulation and Condition (F(1,12) = 1.27, *p* = 0.28) (see Table 2). One potential explanation for the lack of differences between the movie and rest conditions could be due to the effects of rTMS itself, since it has been demonstrated that applying rTMS over frontal regions could impact the autonomic system via the fronto-vagal pathway [41]. The potential rTMS-induced discomfort could also constitute a confounding factor on these measurements given the relationship between pain and autonomic responses [42]. Future studies may wish to compare the effects of frightening movies on electrophysiological measurements without rTMS application to confirm this hypothesis.

According to the state-dependency assumption, it was expected that participants in the active group who experienced higher physiological arousal, as measured by differences in BPM and HRV between movies and resting periods, would show greater changes in amygdala activation. Despite this expectation, the correlation analyses did not reveal any significant relationships between changes in amygdala activation and changes in HRV or BPM.

## 4. Discussion

In this preliminary, proof-of-concept study, connectivity-based rTMS was applied over a node in the mPFC that showed the strongest negative connectivity with the right amygdala, as defined by a PPI analysis. It was anticipated that active rTMS over this target would reduce amygdala activity by strengthening the negative connectivity between these two regions. To improve rTMS efficacy, stimulation was applied online, while subjects passively viewed frightening video clips from the Schaefer et al. database [39], separated by resting periods, under the assumption that engaging the fronto-limbic network with a task while simultaneously stimulating it with rTMS would promote Hebbian-like plasticity.

The results offer a promising suggestive demonstration of the potential for connectivity-based target engagement that is feasible and practical. Interestingly, several results were different from what was expected, as (i) active rTMS did not strengthen negative connectivity between mPFC and amygdala, but instead reversed it—going from a negative to a positive connectivity, (ii) the activity of the indirectly targeted right amygdala activity was increased, instead of decreased, after active relative to sham rTMS, and (iii) no significant changes were observed on ROI-based resting-state connectivity between the left mPFC and the right amygdala.

Understanding how rTMS effects propagate from proximal sites under the coil to distal structures deeper in the brain remains largely unknown. In a recent review article of 33 studies investigating rTMS effects on resting-state functional connectivity [20], it was found that the common rTMS frequency-dependent heuristic observed with proximal brain structures was not typical of studies reporting downstream distal effects, with a majority of studies reporting increased functional connectivity after rTMS, independent of the stimulation frequency. Results from the current study are also discrepant with the common frequency dependent heuristic and point to a more complex relationship between proximal stimulation and downstream connectivity.

Despite this, findings from the current study must be considered in light of several design considerations and limitations. Notably, the current study included a small sample size that indicates feasibility and preliminary effectiveness, but requires larger samples and replication to draw strong conclusions. Secondly, it is important to consider that the observed decrease in amygdala activation occurred between the two visits, and may be attributable to task habituation [40] which could point to either meaningful dishabituation or other uncontrolled confounds. Furthermore, the targeting approach was based on task-related connectivity between the activated amygdala and mPFC, but since the amygdala was not activated by this task during the second MRI acquisition, the mPFC no longer needs to exert inhibitory control over the amygdala. Therefore, it is impossible with these data to define whether the increase in amygdala activation observed between active and sham rTMS during the second visit was due to a connectivity-change or to a change in neural activity in the amygdala. Through continued research it may be possible to specify these relationships. For example, by collecting fMRI before and after rTMS and testing the interaction between stimulation frequency (low versus high frequency) and connectivity profile (stimulating a node positively or negative connected to the amygdala) on amygdala changes. Before implementing these connectivity-based rTMS approaches to treat patients with psychiatric disorders, future studies may also want to investigate how differences in connectivity at baseline affect subsequent rTMS effects. For example, it has been shown that compared to healthy participants, patients with PTSD demonstrate stronger connectivity between the amygdala and the dorsomedial prefrontal cortex when performing an emotional working memory task involving negative pictures from IAPS [43]. Patients with PTSD also show aberrant connectivity at rest [44]. Those differences may lead to opposite rTMS effects and therefore future studies are required to investigate rTMS effects in such patient populations.

## 5. Conclusions

Results from this pilot study indicate the feasibility and promising potential of task-related connectivity-based rTMS on amygdala activation. While this preliminary study is limited by its relatively small sample size and requires replication in larger study samples, the findings point towards important questions and approaches that warrant more scrutiny. Further research will allow us to reliably predict and leverage the down-stream distal effects of rTMS and if successful, these studies could pave the way to more powerful neurotherapeutic approaches that can be applied to patients with fronto-limbic cortical dysregulation, such as those with posttraumatic stress disorders.

## Figures and Tables

**Table 1:** Count of participants’ guess and averaged confidence level in their guess (from 0%: not confident to 100%: definitely confident in their guess) as a function of the true delivered stimulation.

		Participants’ Guess		Confidence Level of Guess	
		Active	Sham	Active	Sham
**Actual Stimulation**:	Active	6	1	79.2	10.0
	Sham	4	2	57.5	27.5

**Table 2:** Averaged beats per minutes (BPM) and heart rate variability (HRV) during movies and resting period for participants who received active or sham rTMS.

	Movies		Rest	
	BPM	HRV	BPM	HRV
**Active rTMS**	65.19 ± 12.88	0.95 ± 0.19	65.39 ± 12.77	0.95 ± 0.19
**Sham rTMS**	68.31 ± 11.59	0.90 ± 0.18	66.17 ± 11.23	0.94 ± 0.17

## Data Availability

Data are available upon request to the corresponding author.
